# A comparability study of natural and deglycosylated PD-L1 levels in lung cancer: evidence from immunohistochemical analysis

**DOI:** 10.1186/s12943-020-01304-4

**Published:** 2021-01-07

**Authors:** Jie Mei, Junying Xu, Xuejing Yang, Dingyi Gu, Weijian Zhou, Huiyu Wang, Chaoying Liu

**Affiliations:** Department of Oncology, Wuxi People’s Hospital Affiliated to Nanjing Medical University, No. 299 Qing Yang Road, Wuxi, 214023 China

## Abstract

Emerging evidence has revealed that the removal of N-linked glycosylation could enhance PD-L1 detection. However, whether PD-L1 antibodies against different epitopes of PD-L1 antigens responding to deglycosylation has not been characterized. In this study, we compared natural and deglycosylated PD-L1 expression in lung cancer (LuCa) using a panel of PD-L1 antibodies (28–8, CAL10, 73–10 and SP142). We found that removal of N-linked glycosylation markedly enhanced PD-L1 detection when the 28–8, CAL10 and SP142 monoclonal antibodies (mAbs) were used but slightly inhibited PD-L1 detection when the 73–10 mAb was used. Moreover, for the CAL10 and SP142 mAbs, deglycosylated PD-L1 levels showed stronger correlations with the response to anti-PD-1 therapy. Overall, our research provides a comprehensive insight into the application of deglycosylated PD-L1 detection, which expands the clinical significance of this established strategy in LuCa.

**Supplementary Information:**

The online version contains supplementary material available at 10.1186/s12943-020-01304-4.

## Main text

Immunotherapy is one of the most encouraging strategies for cancer treatment, and the most common immunotherapy strategy involves the interruption of the interaction between immune checkpoints expressed on tumor and immune cells, which blocks the immune escape of tumor cells to some extent [1]. Programmed-death-ligand 1 (PD-L1) is an important immunosuppressive molecule that is primarily expressed on tumor cells and that has been widely reported across multiple malignant tumors [2]. PD-L1 plays a critical role in triggering the immune escape of cancer by binding to its receptor, PD-1 [3]. The expression status of PD-L1, as detected by immunohistochemistry (IHC) has exhibited a significant correlation with response to immunotherapy, although several limitations of this biomarker exist [4]. Therefore, an improved PD-L1 detection method may be a better guide to immunotherapy in clinical practice.

N-linked glycosylation is a common posttranslational modification of PD-L1, and glycosylated PD-L1 with heavy N-linked glycans has been found in various cancer types and exhibits various patterns on western blots; in contrast, the nonglycosylated form of PD-L1 is detected at ~ 33 kDa [5]. Recently, Lee et al. reported that the removal of N-linked glycosylation could enhance PD-L1 (28–8 clone) detection and more accurately predict the therapeutic efficacy of PD-1/PD-L1 inhibitors [6]. Detection of deglycosylated PD-L1 may therefore be a better biomarker for cancer immunotherapy [7]. However, whether PD-L1 antibodies against different epitopes of PD-L1 antigens responding to glycosylation has not been evaluated.

In this study, we performed a comparability study of natural and deglycosylated PD-L1 expression in lung cancer (LuCa) using a panel of PD-L1 monoclonal antibodies (mAbs) obtained from Abcam. The materials and methods was supplied in Additional file 1: Supplementary materials and methods. As a result, we found that removal of N-linked glycosylation significantly enhanced PD-L1 detection when using the 28–8, CAL10 and SP142 mAbs but slightly inhibited PD-L1 detection when using the 73–10 mAb. In addition, deglycosylated PD-L1 levels determined by the CAL10 and SP142 mAbs showed stronger correlations with the immunotherapeutic response. Overall, our research further expands the clinical significance of deglycosylated PD-L1 detection in LuCa.

## Results and discussion

### Comparability of natural PD-L1 scoring using a panel of PD-L1 antibodies

**Fig. 1:**
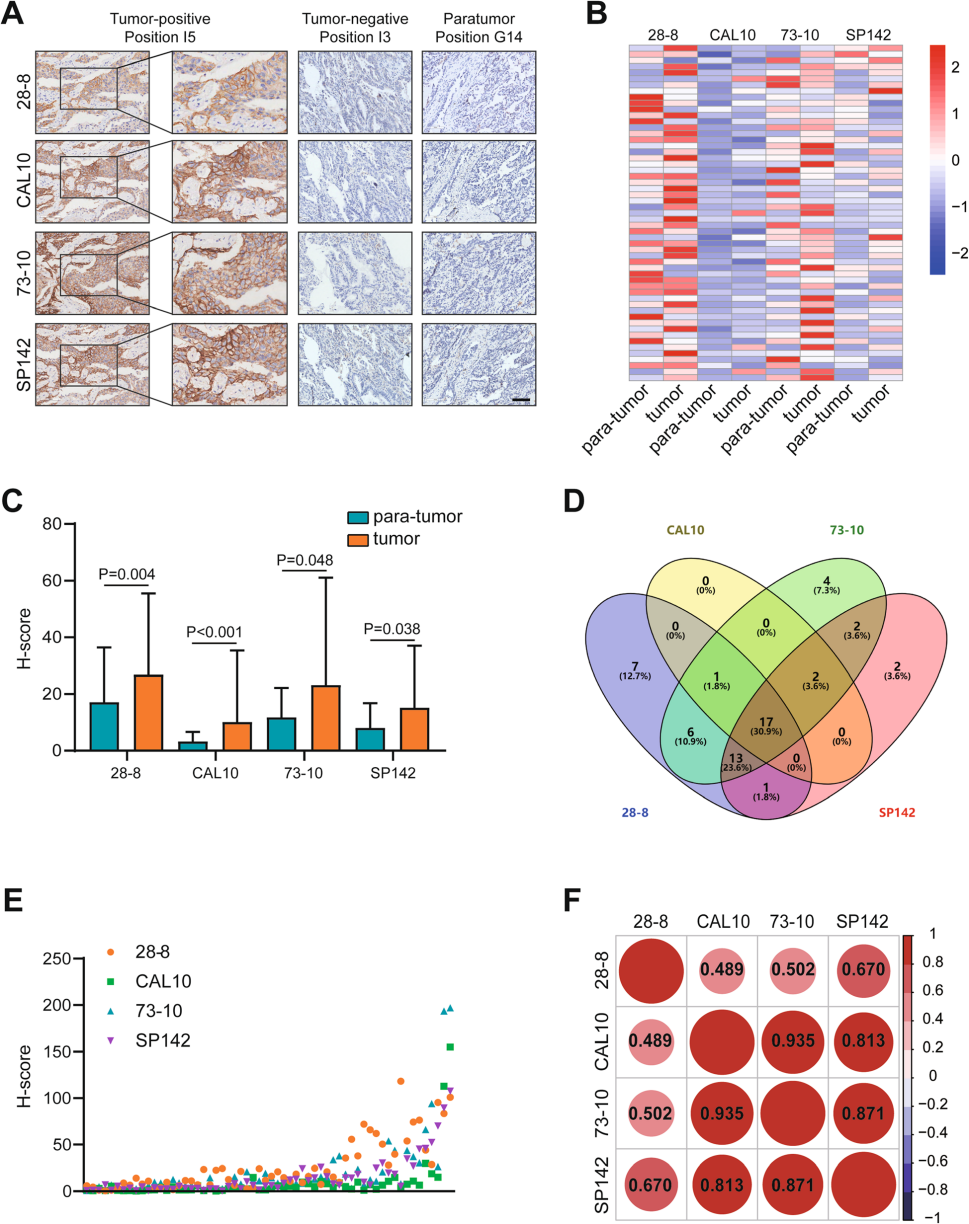
Expression of PD-L1 in LuCa and paratumor tissues and agreement among the 4 mAbs. (A) Representative images show samples stained with each of the 4 PD-L1 mAbs. Bar = 200 μm. (B) Heatmap represents the PD-L1 expression levels in tumor and paratumor tissues after staining with 4 PD-L1 mAbs. Red: high quantitative value, blue: low quantitative value. (C) H-score of PD-L1 signal intensity in tumor and paratumor tissues after staining with 4 PD-L1 mAbs. (D) Venn plot shows the PD-L1 TPS of the 60 cases stained with each of the 4 PD-L1 mAbs and those that scored above and below the threshold of 5%. (E) Data points represent the mean H-score of each sample as calculated by HALO software. Superimposed points indicate identical values. (F) Heatmap represents the correlation of the H-scores after staining with 4 PD-L1 mAbs

The detection of PD-L1 expression status using IHC is the most direct and practicable route for stratification to guide anti-PD-1/PD-L1 therapy [8]. In the current study, we first compared natural PD-L1 expression in LuCa using a panel of antibodies obtained from Abcam, including 28–8, CAL10, 73–10 and SP142. To obtain the best staining effect, we performed IHC at an assay-dependent concentration (Additional file 2: Table S1). The clinicopathological features of LuCa patients represented in the HLugC120PT01 and the array distribution of HLugC120PT01 sections are included in Additional file 3: Table S2 and Additional file 4: Fig. S1. Two paratumor samples exhibited exfoliation of cells, and 3 samples were remarkably infiltrated with tumor cells, which were excluded from this analysis. The representative images exhibited a characteristic PD-L1 staining pattern, which was typified by immunoreactivity mostly in the cytomembrane; besides, the cytoplasm was also partially stained (Fig. 1a). We next compared the PD-L1 expression status in tumor and paratumor tissues. The percentage of PD-L1-positive cells and Histoscore (H-score) of PD-L1 signal intensity in LuCa tissues detected by these 4 mAbs were significantly higher than those in paratumor tissues (Additional file 5: Table S3 and Fig. 1b and c).

To evaluate the agreement among the 4 PD-L1 mAbs, a Venn diagram was generated to compare the percentage of PD-L1-positive tumor cells (tumor proportion score, TPS) in the cohort. Of the 60 cases, five showed concordance below all thresholds of 5% across all mAb combinations, while 17 cases showed expression above all thresholds of 5%. The remaining 38 cases showed a combination of discordant outcomes across the various mAb combinations (Fig. 1d). In addition, we found that the CAL10 mAb had the lowest detection rate (20/60), while the other 3 mAbs had similar detection rates (28–8: 45/60, 73–10: 45/60, SP142: 37/60) in LuCa (Fig. 1d). Using these 4 mAbs, we further analyzed the consistency of PD-L1 with respect to the H-score, and the results revealed high consistency in the detection of PD-L1 expression (Fig. 1e and f). Overall, these 4 PD-L1 mAbs had a similar detection performance in LuCa.

### Comparability of the response to deglycosylation using a panel of PD-L1 antibodies

Lee et al. demonstrated a novel strategy for the detection of PD-L1 expression. Briefly, antigen retrieval by protein deglycosylation could enhance the PD-L1 (28–8 mAb) signal intensity [6]. Due to an increasing number of diagnostic PD-L1 antibodies that have become commercially available, whether the deglycosylation strategy is suitable for antibodies with various antigen specificities is worthy of further exploration. Immunofluorescence staining is an effective method that can detect PD-L1 expression with or without deglycosylation [6]. The fluorescence intensity of PD-L1 determined by three antibodies (28–8, CAL10 and SP142) was significantly enhanced after PNGase F treatment in NCI-H1299 compared with no treatment; however, slight inhibition in the fluorescence intensity of PD-L1 was found after PNGase F treatment with the 73–10 antibody (Additional file 6: Fig. S2).

**Fig. 2:**
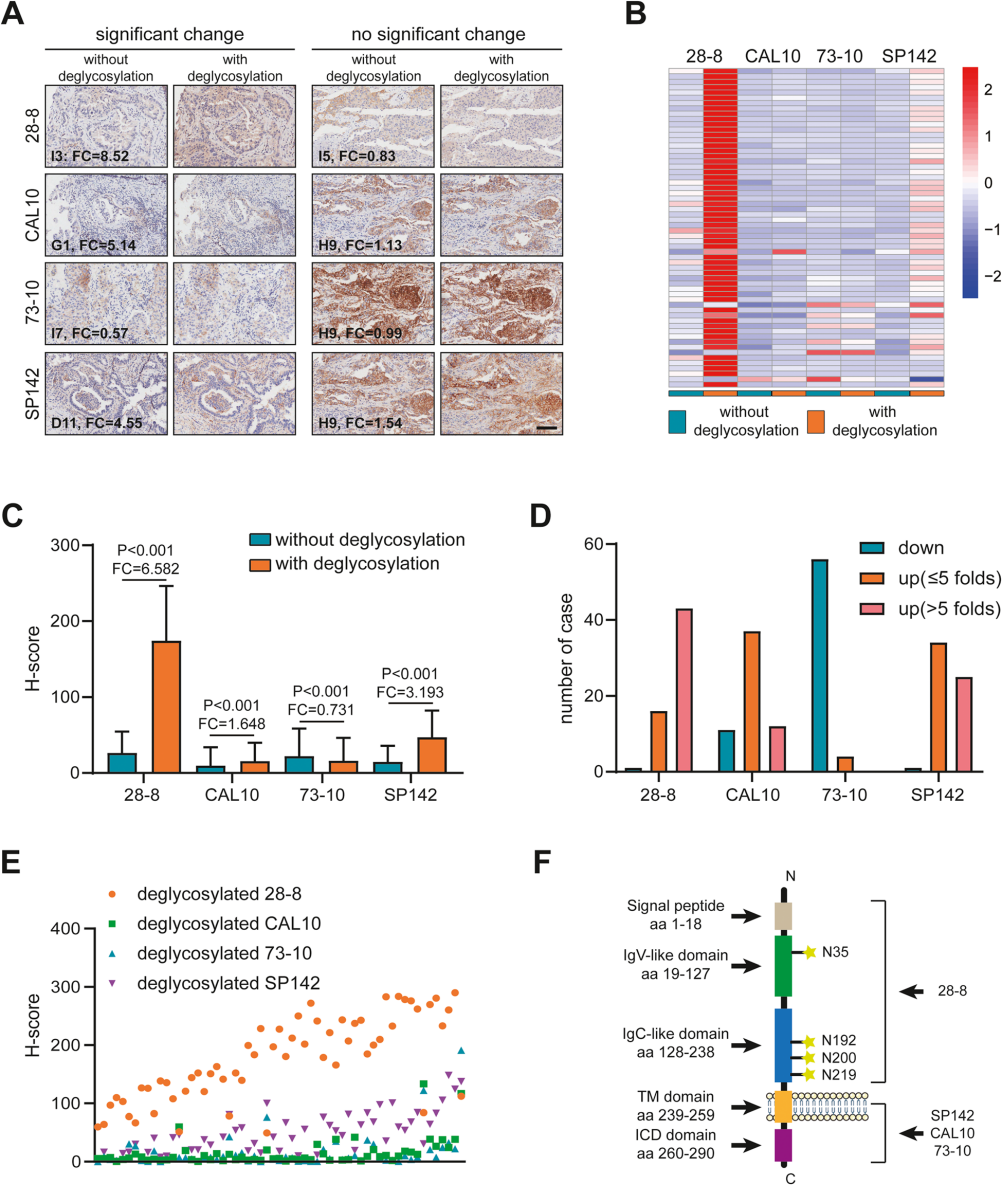
Signal intensity of PD-L1 in LuCa tissues after sample deglycosylation. (A) Representative images show the samples stained with each of the 4 PD-L1 mAbs with or without sample deglycosylation. Bar = 200 μm. (B) Heatmap represents the expression levels of PD-L1 after staining with 4 PD-L1 mAbs with or without sample deglycosylation. Red: high quantitative value, blue: low quantitative value. (C) H-score of PD-L1 signal intensity after staining with 4 PD-L1 mAbs with or without sample deglycosylation. (D) Fold change (FC) of the PD-L1 H-score and the corresponding number of cases. (E) Data points represent the mean H-score of each sample as calculated by HALO software. Superimposed points indicate identical values. (F) The domain structure and glycosylation sites of PD-L1

Next, we evaluated the response of different PD-L1 antibodies to deglycosylation in LuCa tissues. The results showed that removal of N-linked glycosylation markedly enhanced PD-L1 detection when the 28–8, CAL10 and SP142 mAbs were used but slightly inhibited PD-L1 detection when the 73–10 mAb was used (Fig. 2a-d and Additional file 7: Fig. S3). When we compared PD-L1 detection after deglycosylation using these 4 mAbs, the PD-L1 staining level obtained with the 28–8 mAb was the highest, while the level obtained with SP142 mAb was close behind (Fig. 2e). In addition, after deglycosylation, PD-L1 expression was not limited to the tumor cell membrane but could also be detected in the cytoplasm (Fig. 2a). Several studies have reported that PD-L1 can also be expressed in the tumor cell cytoplasm [9, 10]. According to the report by Lee et al., PD-L1 detection was significantly enhanced in both the cell membrane and cytoplasm, which was consistent with our results [6].

According to specifications provided by Abcam, the peptides used for the production of the CAL10, SP142 and 73–10 mAbs are generated against the intracellular domain (the exact sequence is not available), and the peptide used for production of the 28–8 mAb is generated against the extracellular domain (Phe19-Thr239). Four glycosylation sites have been previously reported, including N35, N192, N200 and N219, which are all located in the extracellular domain of PD-L1 [5] (Fig. 2f). Thus, the PD-L1 signal intensity could be significantly enhanced when using the 28–8 mAb since the PD-L1 antigenic region is more accessible to antibody binding. However, although the exact glycosylation sites in the intracellular domain were not available, the PD-L1 signal intensity could also be enhanced to some extent when the CAL10 and SP142 mAbs were used. We speculated that the adjacent glycosylation sites, such as N200 and/or N219, reduced the binding affinity. Regardless, our current results showed that sample deglycosylation can not only enhance the 28–8 mAb signal but also improve the detection rate of the CAL10 and SP142 mAbs.

### Associations between response to deglycosylation and efficacy of anti-PD-1 therapy

**Fig. 3:**
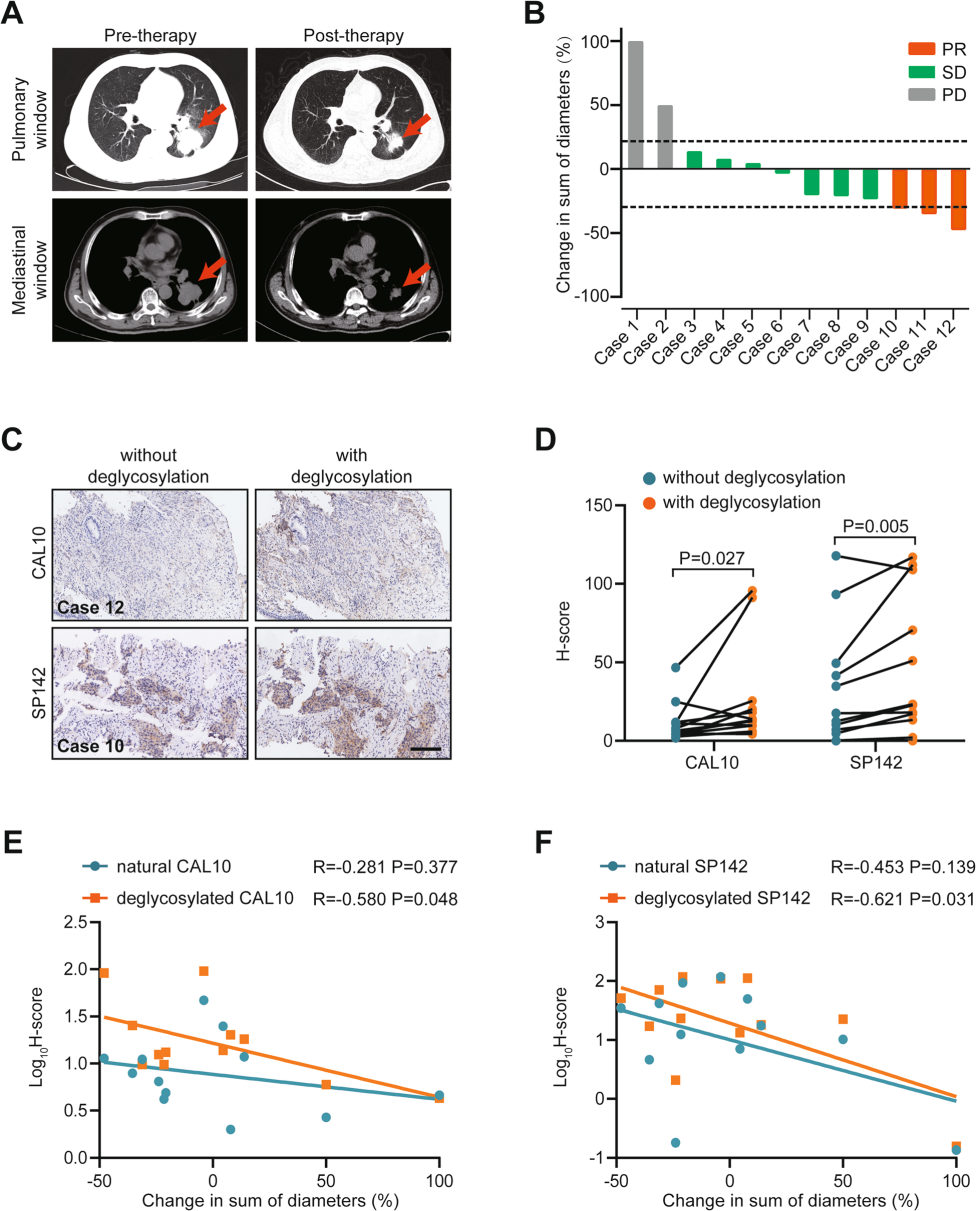
Associations between deglycosylated PD-L1 levels and response to anti-PD-1 therapy. (A) Representative CT images show one patient who derived a substantial benefit from camrelizumab. (B) Overview of the relative change in sum of diameters in the recruited cohort. (C) Representative images show samples from the recruited cohort that were stained with each of the 2 PD-L1 mAbs (CAL10 and SP142) with or without sample deglycosylation. Bar = 200 μm. (D) H-score of PD-L1 signal intensity after staining with 2 PD-L1 mAbs with or without sample deglycosylation. (E, F) Correlation between the H-score of PD-L1 after staining with CAL10 and SP142 mAbs before and after sample deglycosylation and the relative change in sum of diameters

Furthermore, to test whether the deglycosylated PD-L1 level would better predict the response to immunotherapy, 12 LuCa patients who received anti-PD-1 therapy (camrelizumab plus chemotherapy) were recruited (Additional file 8: Table S4). One representative patient who derived a substantial benefit from camrelizumab therapy is shown in Fig. 3a. Besides, the relative change in sum of diameters was exhibited in Fig. 3b. Given the better predictive value of deglycosylated PD-L1 determined by the 28–8 mAb in immunotherapy uncovered by Lee et al. [6], we did not include it in current research. At first, we validated that tissue deglycosylation could enhance the detection rate of the CAL10 and SP142 mAbs in our recruited cohort (Fig. 3c and d). Encouragingly, the relative change in sum of diameters exhibited a stronger correlation with deglycosylated PD-L1 than with natural PD-L1 levels determined by the CAL10 and SP142 mAbs, which suggested that deglycosylated PD-L1 expression might be a better biomarker for predicting efficacy to at least camrelizumab-based therapies but potentially other anti-PD-1 therapies as well (Fig. 3e and f). However, the clinical significance of deglycosylated PD-L1 should be further explored in large-scale clinical trials. Studies should include at least the following three critical points: 1. standard technical protocol for deglycosylated PD-L1 detection, 2. cutoff values of deglycosylated PD-L1 levels for use in clinical practice, and 3. the congruent relationship between different detection antibodies and therapeutic antibodies.

## Conclusions

Due to the significance of sample deglycosylation, we systematically investigated the impact of the deglycosylation strategy using 4 PD-L1 mAbs. The removal of N-linked glycosylation from PD-L1 in tissue samples can enhance 28–8, CAL10 and SP142 mAbs-based detection. In addition, deglycosylated PD-L1 levels determined by the CAL10 and SP142 mAbs showed stronger correlations with the response to immunotherapy. In conclusion, sample deglycosylation can provide a more precise estimation of PD-L1 levels to reduce false-negative results in clinical practice.

## Supplementary Information

**Additional file 1 Supplementary materials and methods**

**Additional file 2.** Table S1. Dilution ratio and antigen retrieval reagents used for PD-L1 antibodies

**Additional file 3.** Table S2. Clinicopathological features of LuCa patients in HLugC120PT01 section

**Additional file 4 Fig. S1**. The array distribution of HLugC120PT01

**Additional file 5.** Table S3. Comparison of positive rate of PD-L1 between LuCa and para-tumor tissues

**Additional file 6 Fig. S2**. Signal intensity of PD-L1 in NCI-H1299 LuCa cells after sample deglycosylation

**Additional file 7 Fig. S3**. Distribution of fold change (FC) values of PD-L1 signal intensity stained by 4 mAbs with or without sample deglycosylation

**Additional file 8.** Table S4. Detailed clinical information of the recruited LuCa patients

## Data Availability

All data generated or analyzed during this study are included in this published article and its additional files.

## Abbreviations

PD-L1: programmed-death-ligand 1
IHC: immunohistochemistry
LuCa: lung cancer
mAbs: monoclonal antibodies
H-score: Histoscore
TPS: tumor proportion score
