# Supplementary material for: A comparability study of natural and deglycosylated PD-L1 levels in lung cancer: evidence from immunohistochemical analysis
Source: Mol Cancer. 2021 Jan 7;20:11. doi: 10.1186/s12943-020-01304-4 (PMC7789157; doi:10.1186/s12943-020-01304-4)
Supplement: Supplementary file 1 — Additional file 1 Supplementary materials and methods [file 12943_2020_1304_MOESM1_ESM.docx]

**Supplementary materials and methods**

**Reagents and materials**

The panel of PD-L1 antibodies (28-8, 73-10, SP142 and CAL10) and the critical reagents for 28-8 clone PD-L1 immunohistochemistry (IHC), including the heat-induced epitope retrieval (HIER) reagent (ab208572) and the IHC detection kit HRP/DAB (ab209101), were obtained from Abcam. Recombinant PNGase F (P0708) was obtained from New England Biolabs. Conventional reagents used for IHC staining were obtained from Dako unless otherwise noted. A high-density lung cancer (LuCa) tissue microarray (TMA) with serial sections (HLugC120PT01) was obtained from Outdo Biotech and contained 60 LuCa samples of different histological types and paired paratumor specimens. The TMA was accompanied by integrated clinicopathological information (Additional file 3: Table S2).

**Collection of clinical samples**

A total of 12 LuCa patients who received anti-PD-1 therapy (camrelizumab plus chemotherapy) were recruited by the Wuxi People’s Hospital affiliated to Nanjing Medical University from 2019 to 2020. The assessment was performed every 6 weeks or whenever the patients showed obviously advanced symptoms. The evaluation was independently conducted by two oncologists according to RECIST criterion: complete response (CR), partial response (PR), stable disease (SD) and progressive disease (PD). Detailed clinicopathological information is shown in Additional file 8: Table S4. Correlations between the relative change in sum of diameters and the log_10_(PD-L1 H-score) transformed value were assessed by Pearson analysis. Ethical approval for the current study was granted by the Clinical Research Ethics Committee, Wuxi People’s Hospital affiliated to Nanjing Medical University.

**Cell deglycosylation and immunofluorescence confocal microscopy**

NCI-H1299 cell line with low PD-L1 expression was purchased from KeyGEN BioTECH *Inc.* (Nanjing, China) and maintained in RPMI-1640 (KeyGEN BioTECH *Inc.*) supplemented with 10% (v/v) fetal bovine serum (Gibco) at 37°C with 5% CO_2_. To perform cell deglycosylation, LuCa cells seeded in 35-mm dish were fixed in 4% paraformaldehyde at 4°C overnight. Similar to the report by Lee [1], after they were washed three times with PBS, the fixed cells were incubated with 1× glycoprotein denaturing buffer, denatured by heating at 100°C for 10 min, and chilled on ice. The denaturing buffer was removed from the chamber, and the cells were washed with PBS three times, treated with or without 5% PNGase F dissolved in glycosylated buffer (20% of 10× GlycoBuffer 2, 20% of 10% Nonidet P-40, and 60% H_2_O) at 37°C for 12 hr. The cells were then subjected to immunofluorescence and confocal microscopy using PD-L1 antibodies (28-8, 1:500 dilution; CAL10, 1:500 dilution; 73-10, 1:200 dilution; SP142, 1:50 dilution). Images were captured by a Zeiss LSM710 laser scanning confocal microscope.

**Tissue deglycosylation and immunohistochemistry**

Tissue deglycosylation and IHC staining were performed directly on the TMAs and collected sections. Sections were incubated at 40°C overnight and then at 60°C for 1 hr, deparaffinized in xylene and rehydrated in ethanol and distilled water. HIER was performed with different retrieval reagents according to the protocols provided by Abcam (Table S1). In contrast to Lee’s report [1], to thoroughly remove the N-linked glycans of PD-L1, we applied the thermal denaturation strategy. Briefly, after washing with PBS three times, TMA sections were incubated with 1× glycoprotein denaturing buffer at 95°C for 5 min, washed with PBS three times, treated with or without 5% recombinant PNGase F (P0708, New England Biolabs) dissolved in glycosylated buffer (20% of 10× GlycoBuffer 2, 20% of 10% Nonidet P-40, and 60% H_2_O) at 37°C for 1 hr, and then subjected to IHC. TMAs were incubated with anti-PD-L1 antibodies overnight at 4°C. The primary antibodies against PD-L1 used in the current study are shown in Table S1. Antibody staining was visualized with DAB and hematoxylin counterstain. Immunostained sections were scanned using Aperio Digital Pathology Slide Scanners.

**Quantification of PD-L1 level**

The imaging and quantification of IHC staining was performed with HALO Next Generation Imaging analysis software (Indica Labs). HALO measures and reports individual cell data and maintains an interactive link between cell metrics and cell imagery. All staining procedures performed resulted in a characteristic tumor cell pattern of PD-L1 membrane staining. The results of IHC were evaluated using an established semiquantitative approach that assessed the percentage of positively stained tumor cells (tumor proportion score, TPS) or the percentage of positively stained epithelial cells as well as the Histoscore (H-score). The H-score accounted for both the staining intensity and the the percentage of positively stained cells [2]. The H-score was calculated using the following formula: H-score = [1 × (% of cells stained in intensity category 1) + 2 × (% of cells stained in intensity category 2) + 3 × (% of cells stained in intensity category 3)]. The final H-score, which ranged from 0 to 300, was obtained for each stained slide.

**Statistical analysis**

Statistical analyses and visualization were performed using GraphPad Prism 8.0 and R software 4.0.2. All error bars denote standard deviation. Wilcoxon matched-pairs signed rank test was used to compare the difference between two groups, including the H-score between tumor and paratumor tissues as well as before and after deglycosylation. Pearson correlation test were used to determine the linear correlation between two variables. Pearson’s chi-squared test was used to compare the positive rate of PD-L1 between LuCa and paratumor tissues. A two-tailed P value ≤ 0.05 was considered statistically significant.

**References**

1. Lee HH, Wang YN, Xia W, Chen CH, Rau KM, Ye L, et al. Removal of N-Linked Glycosylation Enhances PD-L1 Detection and Predicts Anti-PD-1/PD-L1 Therapeutic Efficacy. Cancer Cell. 2019;36(2):168-78 e4.

2. Detre S, Saclani Jotti G, Dowsett M. A "quickscore" method for immunohistochemical semiquantitation: validation for oestrogen receptor in breast carcinomas. J Clin Pathol. 1995;48(9):876-8.
