# Supplementary material for: A comparability study of natural and deglycosylated PD-L1 levels in lung cancer: evidence from immunohistochemical analysis
Source: Mol Cancer. 2021 Jan 7;20:11. doi: 10.1186/s12943-020-01304-4 (PMC7789157; doi:10.1186/s12943-020-01304-4)
Supplement: Supplementary file 2 — Additional file 2. Table S1. Dilution ratio and antigen retrieval reagents used for PD-L1 antibodies [file 12943_2020_1304_MOESM2_ESM.docx]

Table S1. Dilution ratio and antigen retrieval reagents used for PD-L1 antibodies.

| mAbs | Dil. | Antigen retrieval reagents |
| --- | --- | --- |
| 28-8 (ab205921, Abcam) | 1:500 | HIER buffer (ab208572, Abcam) |
| CAL10 (ab237726, Abcam) | 1:500 | sodium citrate buffer (K8005, Dako) |
| 73-10 (ab228415, Abcam) | 1:500 | EDTA buffer (K8004, Dako) |
| SP142 (ab228462, Abcam) | 1:50 | EDTA buffer (K8004, Dako) |

Note: mAb: monoclonal antibody; dil.: dilution ratio, using at an assay-dependent concentration.
