# Supplementary material for: A comparability study of natural and deglycosylated PD-L1 levels in lung cancer: evidence from immunohistochemical analysis
Source: Mol Cancer. 2021 Jan 7;20:11. doi: 10.1186/s12943-020-01304-4 (PMC7789157; doi:10.1186/s12943-020-01304-4)
Supplement: Supplementary file 3 — Additional file 3. Table S2. Clinicopathological features of LuCa patients in HLugC120PT01 section [file 12943_2020_1304_MOESM3_ESM.docx]

Table S2. Clinicopathological features of LuCa patients in HLugC120PT01 section.

| Clinicopathological factors | Case | Proportion (%) |
| --- | --- | --- |
| Gender |  |  |
| Female | 18 | 30.00% |
| Male | 42 | 70.00% |
| Age |  |  |
| ≤60 | 33 | 55.00% |
| >60 | 27 | 45.00% |
| Tumor size |  |  |
| <5cm | 39 | 65.00% |
| ≥5cm | 21 | 35.00% |
| Lymphatic metastasis |  |  |
| N0 | 10 | 16.67% |
| N1-3 | 57 | 95.00% |
| Unknown | 3 | 5.00% |
| Differentiation degree |  |  |
| Well | 33 | 55.00% |
| Poor | 22 | 36.67% |
| Unknown | 5 | 8.33% |
| Histological type |  |  |
| Large cell carcinoma | 5 | 8.33% |
| Squamous cell carcinoma | 14 | 23.33% |
| Adenocarcinoma | 26 | 43.33% |
| Adenosquamous cell carcinoma | 7 | 11.67% |
| Small cell carcinoma | 8 | 13.33% |

Note: LuCa: lung cancer.
