# Supplementary figures and images for: A comparability study of natural and deglycosylated PD-L1 levels in lung cancer: evidence from immunohistochemical analysis
Source: Mol Cancer. 2021 Jan 7;20:11. doi: 10.1186/s12943-020-01304-4 (PMC7789157; doi:10.1186/s12943-020-01304-4)

**
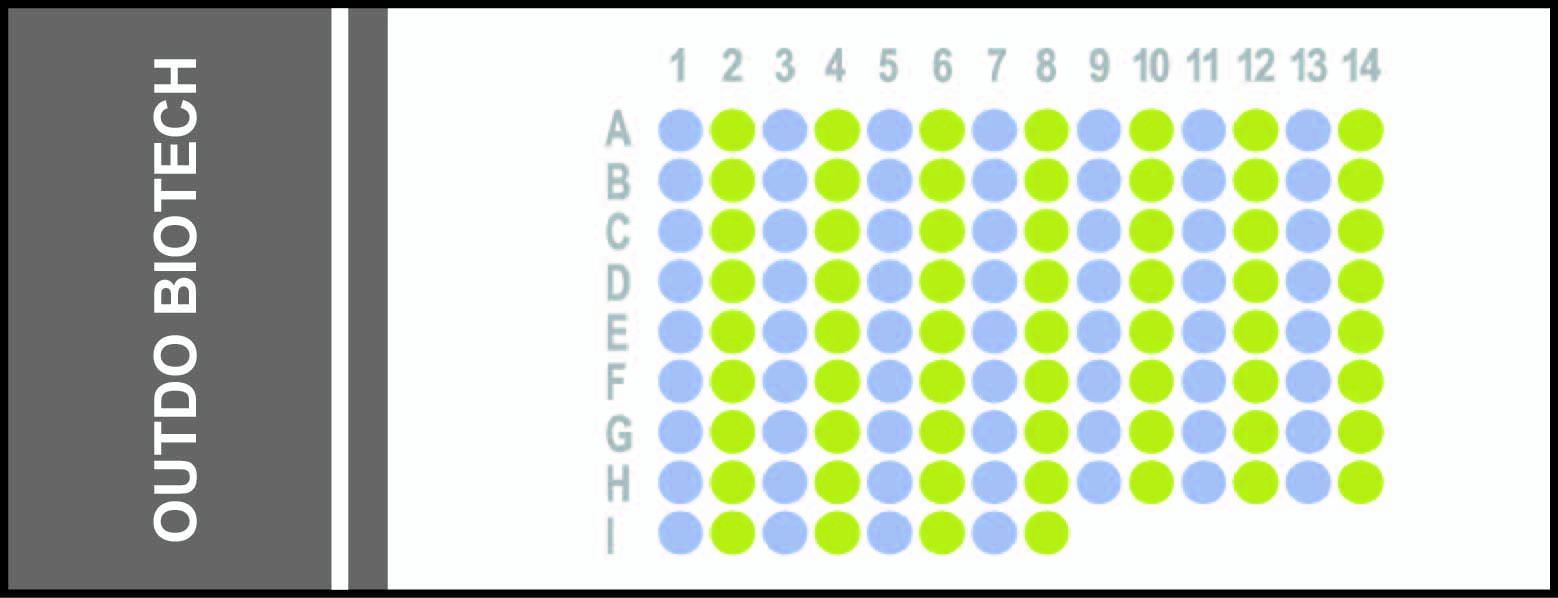
**

**Figure S1. The array distribution of HLugC120PT01.**

Supplement: Supplementary file 4 — Additional file 4 Fig. S1. The array distribution of HLugC120PT01 [file 12943_2020_1304_MOESM4_ESM.docx]
