# Supplementary material for: A comparability study of natural and deglycosylated PD-L1 levels in lung cancer: evidence from immunohistochemical analysis
Source: Mol Cancer. 2021 Jan 7;20:11. doi: 10.1186/s12943-020-01304-4 (PMC7789157; doi:10.1186/s12943-020-01304-4)
Supplement: Supplementary file 5 — Additional file 5. Table S3. Comparison of positive rate of PD-L1 between LuCa and para-tumor tissues [file 12943_2020_1304_MOESM5_ESM.docx]

Table S3. Comparison of positive rate of PD-L1 between LuCa and para-tumor tissues.

| mAbs | PD-L1 status* | Tumor tissue | Para-tumor tissue | χ^2^ | P value |
| --- | --- | --- | --- | --- | --- |
| 28-8 | Positive | 42 (76.36%) | 32 (58.18%) | 4.129 | 0.042 |
|  | Negative | 13 (23.64%) | 23 (41.82%) |  |  |
| CAL10 | Positive | 19 (34.55%) | 6 (10.91%) | 8.748 | 0.003 |
|  | Negative | 36 (65.45%) | 49 (89.09%) |  |  |
| 73-10 | Positive | 41 (74.55%) | 25 (45.45%) | 4.807 | 0.028 |
|  | Negative | 14 (25.45%) | 30 (54.55%) |  |  |
| SP142 | Positive | 34 (61.82%) | 23 (41.82%) | 4.406 | 0.036 |
|  | Negative | 21 (38.18%) | 32 (58.18%) |  |  |

Note: LuCa: lung cancer. *The threshold of positive PD-L1 status was defined as the percentage of positively stained tumor or epithelial cells ≥ 5%.
