# Supplementary material for: A comparability study of natural and deglycosylated PD-L1 levels in lung cancer: evidence from immunohistochemical analysis
Source: Mol Cancer. 2021 Jan 7;20:11. doi: 10.1186/s12943-020-01304-4 (PMC7789157; doi:10.1186/s12943-020-01304-4)
Supplement: Supplementary file 6 — Additional file 6 Fig. S2. Signal intensity of PD-L1 in NCI-H1299 LuCa cells after sample deglycosylation [file 12943_2020_1304_MOESM6_ESM.docx]

**
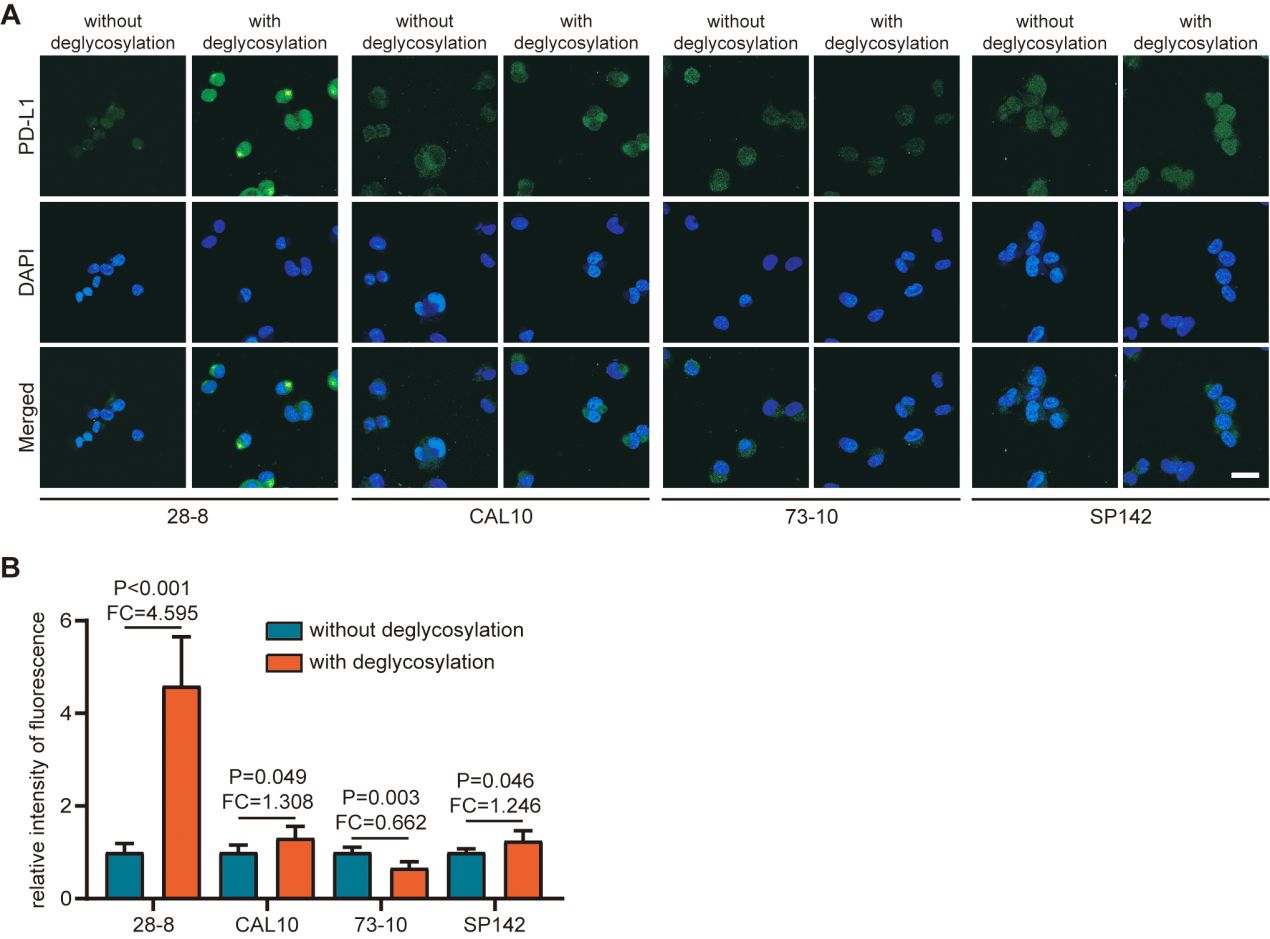
Figure S2. Signal intensity of PD-L1 in NCI-H1299 LuCa cells after sample deglycosylation.** (A) Representative images shows the samples stained with each of the 4 PD-L1 mAbs with or without sample deglycosylation. Bar=30 μm. (B) Intensity of PD-L1 fluorescence stained by 4 PD-L1 mAbs with or without sample deglycosylation.
