# Supplementary material for: A comparability study of natural and deglycosylated PD-L1 levels in lung cancer: evidence from immunohistochemical analysis
Source: Mol Cancer. 2021 Jan 7;20:11. doi: 10.1186/s12943-020-01304-4 (PMC7789157; doi:10.1186/s12943-020-01304-4)
Supplement: Supplementary file 7 — Additional file 7 Fig. S3. Distribution of fold change (FC) values of PD-L1 signal intensity stained by 4 mAbs with or without sample deglycosylation [file 12943_2020_1304_MOESM7_ESM.docx]

**
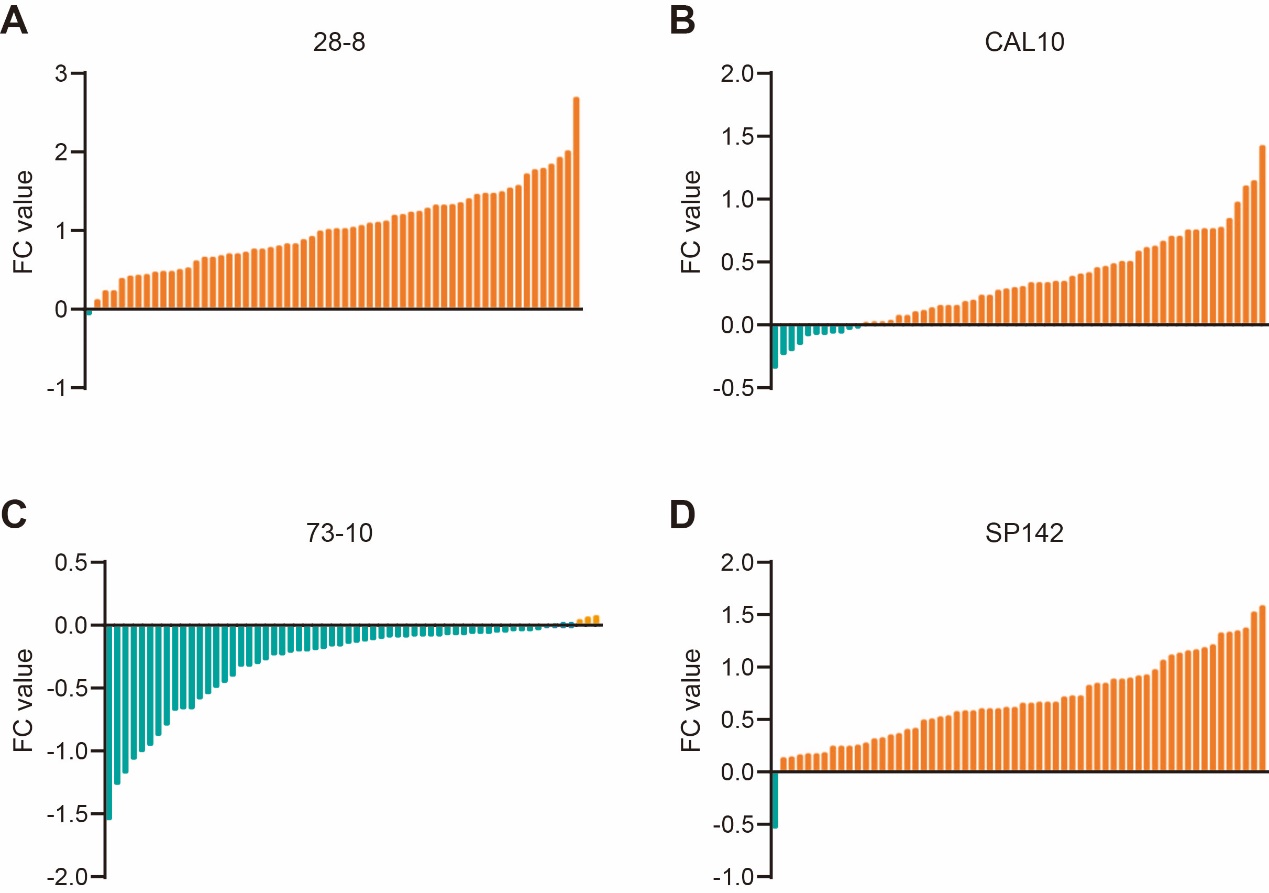
**

**Figure S3. Distribution of fold change (FC) values** **of PD-L1 signal intensity stained by 4 mAbs with or without sample deglycosylation.** (A) 28-8. (B) CAL10. (C) 73-10. (D) SP142.
