# Supplementary material for: A comparability study of natural and deglycosylated PD-L1 levels in lung cancer: evidence from immunohistochemical analysis
Source: Mol Cancer. 2021 Jan 7;20:11. doi: 10.1186/s12943-020-01304-4 (PMC7789157; doi:10.1186/s12943-020-01304-4)
Supplement: Supplementary file 8 — Additional file 8. Table S4. Detailed clinical information of the recruited LuCa patients [file 12943_2020_1304_MOESM8_ESM.docx]

Table S4. Detailed clinical information of the recruited LuCa patients.

| Case | Gender | Age | Histological type | TNM stage | Relative change in sum of diameters | Response evaluation |
| --- | --- | --- | --- | --- | --- | --- |
| Case 1 | Male | 62 | Adenocarcinoma | IV | 100.00% | PD# |
| Case 2 | Male | 71 | Adenocarcinoma | IV | 50.00% | PD* |
| Case 3 | Male | 71 | Adenocarcinoma | IV | 13.89% | SD |
| Case 4 | Male | 51 | Adenocarcinoma | IV | 7.84% | SD |
| Case 5 | Male | 74 | Adenocarcinoma | IV | 4.55% | SD |
| Case 6 | Male | 74 | Adenocarcinoma | IV | -3.94% | SD |
| Case 7 | Male | 63 | Adenocarcinoma | IV | -20.69% | SD |
| Case 8 | Male | 63 | Adenocarcinoma | IV | -21.57% | SD |
| Case 9 | Male | 71 | Adenocarcinoma | IV | -23.91% | SD |
| Case 10 | Male | 64 | Adenocarcinoma | IV | -31.11% | PR |
| Case 11 | Male | 73 | Adenocarcinoma | IV | -35.43% | PR |
| Case 12 | Male | 53 | Adenocarcinoma | IV | -47.92% | PR |

Note: LuCa: lung cancer; PR: partial response; SD: stable disease; PD: progressive disease. #distant metastasis; *new lesions.
